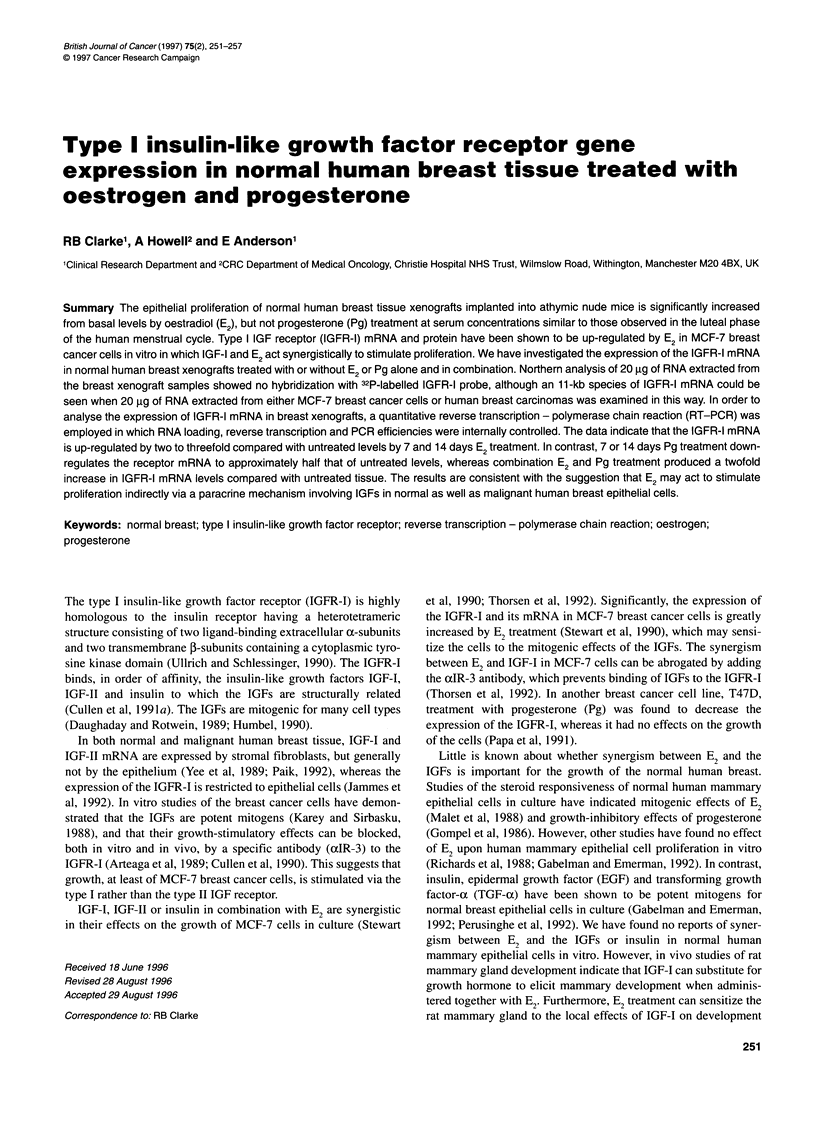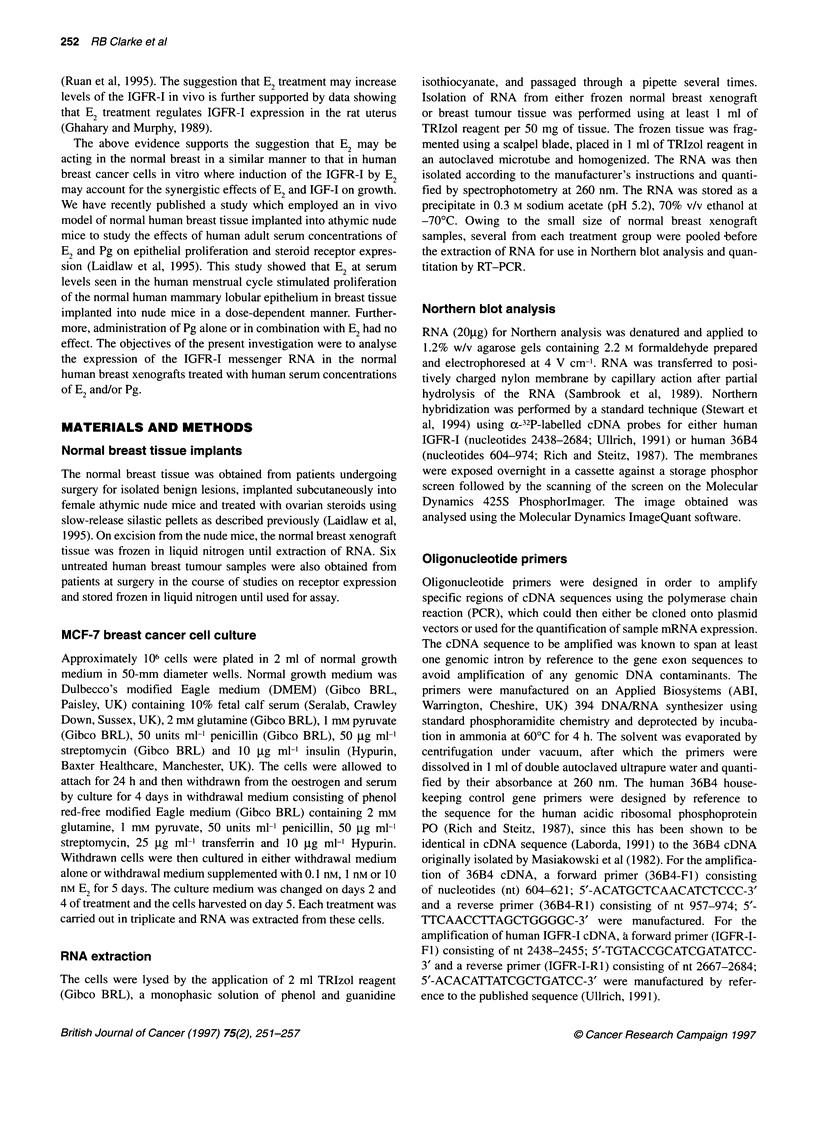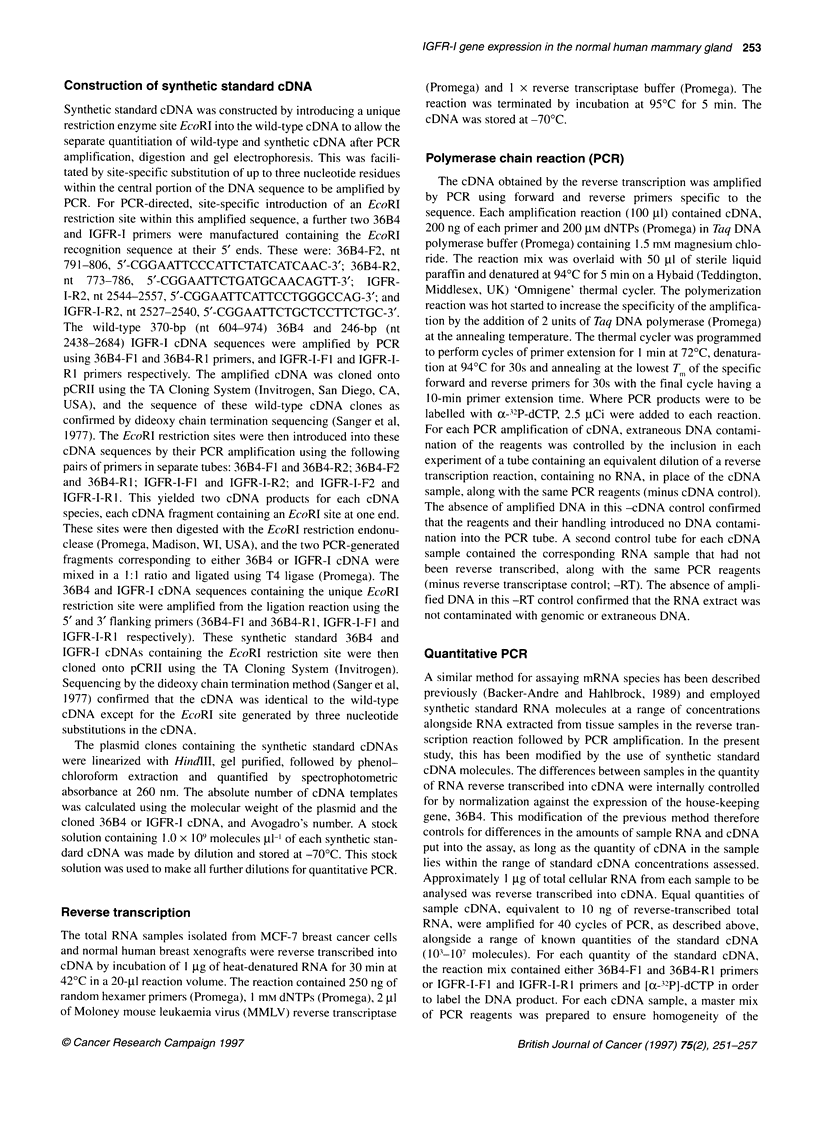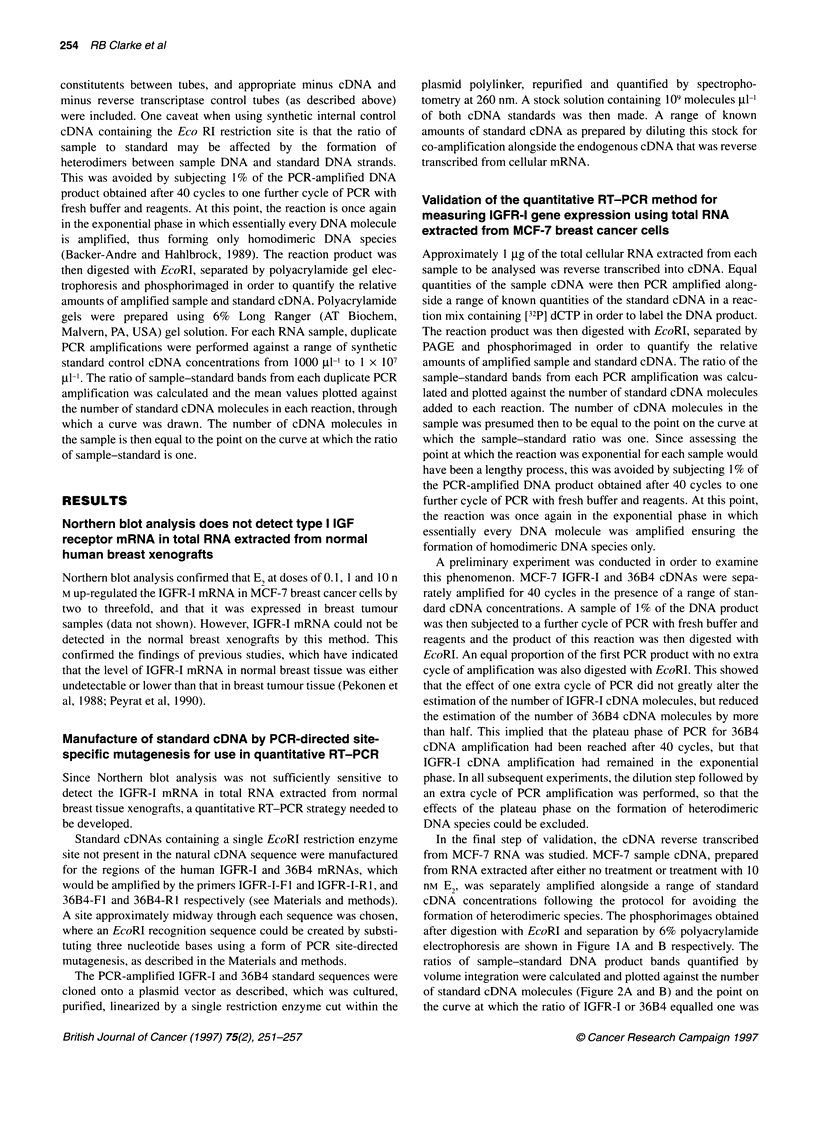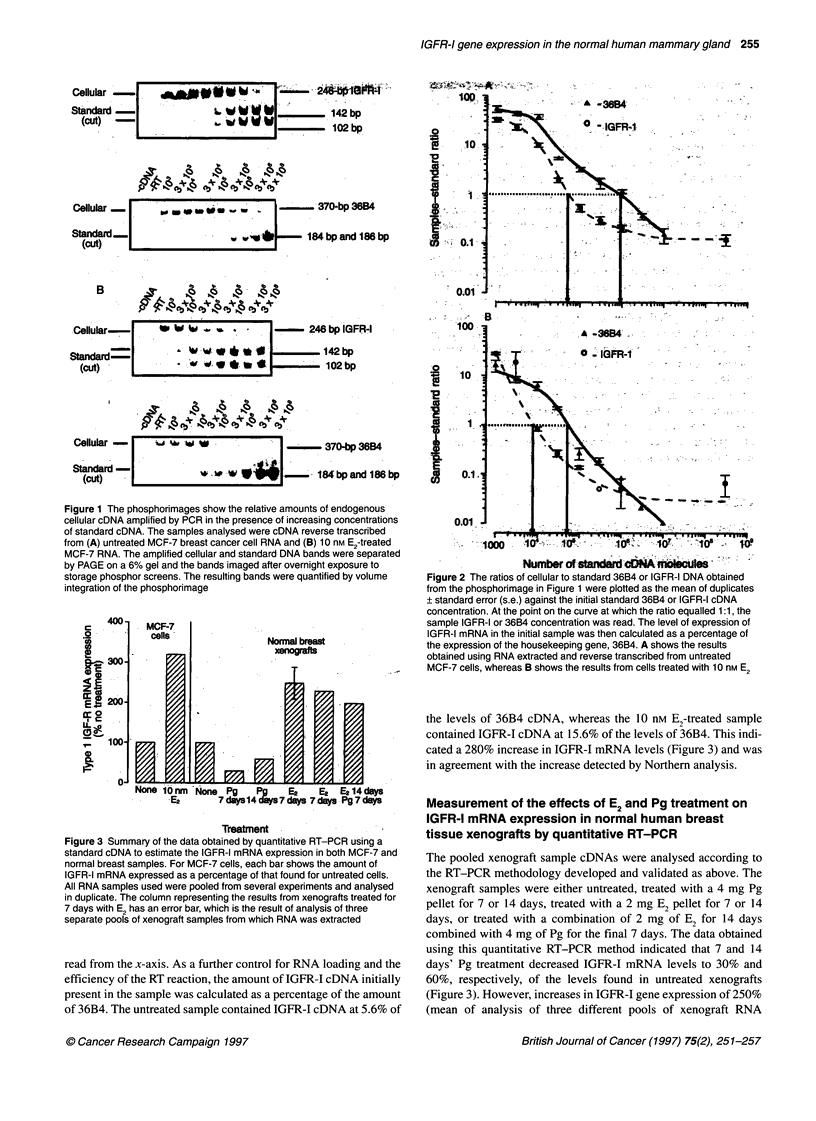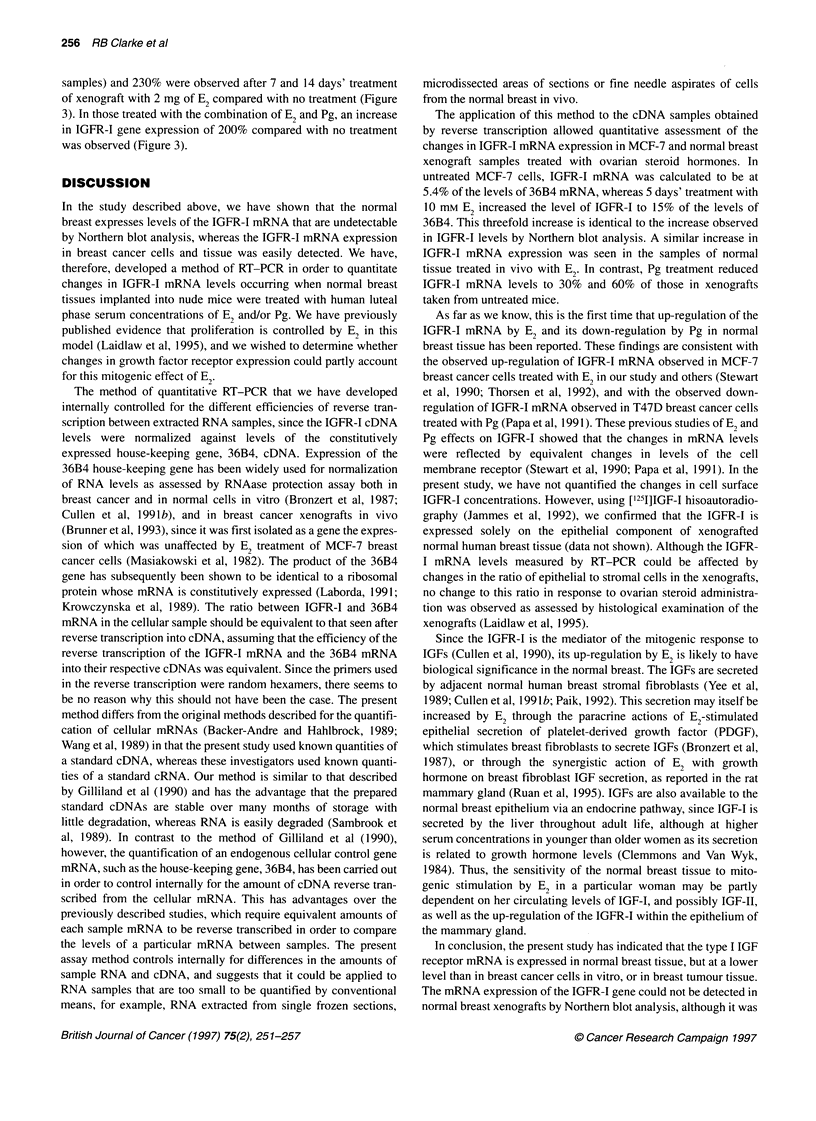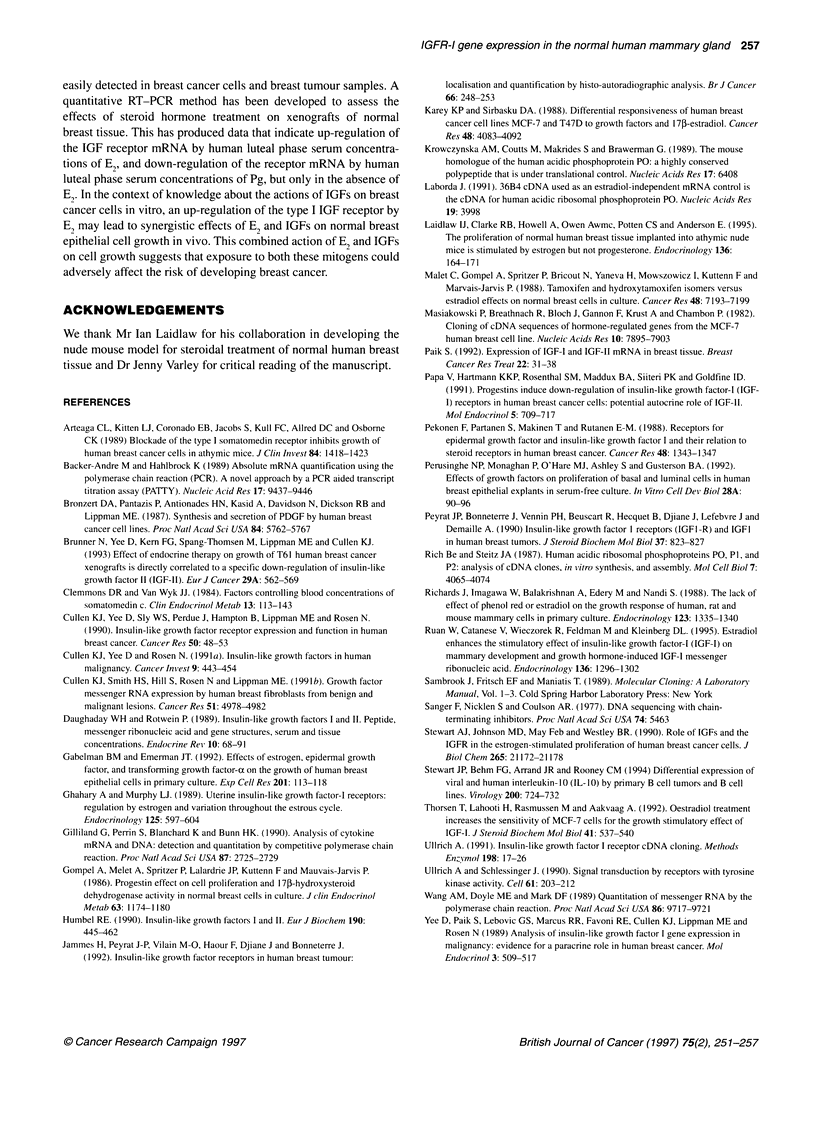# Type I insulin-like growth factor receptor gene expression in normal human breast tissue treated with oestrogen and progesterone.

**DOI:** 10.1038/bjc.1997.41

**Published:** 1997

**Authors:** R. B. Clarke, A. Howell, E. Anderson

**Affiliations:** Clinical Research Department, Christie Hospital NHS Trust, Withington, Manchester, UK.

## Abstract

The epithelial proliferation of normal human breast tissue xenografts implanted into athymic nude mice is significantly increased from basal levels by oestradiol (E2), but not progesterone (Pg) treatment at serum concentrations similar to those observed in the luteal phase of the human menstrual cycle. Type I IGF receptor (IGFR-I) mRNA and protein have been shown to be up-regulated by E2 in MCF-7 breast cancer cells in vitro in which IGF-I and E2 act synergistically to stimulate proliferation. We have investigated the expression of the IGFR-I mRNA in normal human breast xenografts treated with or without E2 or Pg alone and in combination. Northern analysis of 20 micrograms of RNA extracted from the breast xenograft samples showed no hybridization with 32P-labelled IGFR-I probe, although an 11-kb species of IGFR-I mRNA could be seen when 20 micrograms of RNA extracted from either MCF-7 breast cancer cells or human breast carcinomas was examined in this way. In order to analyse the expression of IGFR-I mRNA in breast xenografts, a quantitative reverse transcription-polymerase chain reaction (RT-PCR) was employed in which RNA loading, reverse transcription and PCR efficiencies were internally controlled. The data indicate that the IGFR-I mRNA is up-regulated by two to threefold compared with untreated levels by 7 and 14 days E2 treatment. In contrast, 7 or 14 days Pg treatment down-regulates the receptor mRNA to approximately half that of untreated levels, whereas combination E2 and Pg treatment produced a twofold increase in IGFR-I mRNA levels compared with untreated tissue. The results are consistent with the suggestion that E2 may act to stimulate proliferation indirectly via a paracrine mechanism involving IGFs in normal as well as malignant human breast epithelial cells.


British Journal of Cancer (1997) 75(2), 251–257
© 1997 Cancer Research Campaign

### Type I insulin-like growth factor receptor gene expression in normal human breast tissue treated with oestrogen and progesterone

**RB Clarke[1], A Howell[2] and E Anderson[1]**

[1]Clinical Research Department and [2]CRC Department of Medical Oncology, Christie Hospital NHS Trust, Wilmslow Road, Withington, Manchester M20 4BX, UK

**Summary** The epithelial proliferation of normal human breast tissue xenografts implanted into athymic nude mice is significantly increased from basal levels by oestradiol ($E_2$), but not progesterone (Pg) treatment at serum concentrations similar to those observed in the luteal phase of the human menstrual cycle. Type I IGF receptor (IGFR-I) mRNA and protein have been shown to be up-regulated by $E_2$ in MCF-7 breast cancer cells in vitro in which IGF-I and $E_2$ act synergistically to stimulate proliferation. We have investigated the expression of the IGFR-I mRNA in normal human breast xenografts treated with or without $E_2$ or Pg alone and in combination. Northern analysis of 20 μg of RNA extracted from the breast xenograft samples showed no hybridization with $^{32}$P-labelled IGFR-I probe, although an 11-kb species of IGFR-I mRNA could be seen when 20 μg of RNA extracted from either MCF-7 breast cancer cells or human breast carcinomas was examined in this way. In order to analyse the expression of IGFR-I mRNA in breast xenografts, a quantitative reverse transcription – polymerase chain reaction (RT–PCR) was employed in which RNA loading, reverse transcription and PCR efficiencies were internally controlled. The data indicate that the IGFR-I mRNA is up-regulated by two to threefold compared with untreated levels by 7 and 14 days $E_2$ treatment. In contrast, 7 or 14 days Pg treatment down-regulates the receptor mRNA to approximately half that of untreated levels, whereas combination $E_2$ and Pg treatment produced a twofold increase in IGFR-I mRNA levels compared with untreated tissue. The results are consistent with the suggestion that $E_2$ may act to stimulate proliferation indirectly via a paracrine mechanism involving IGFs in normal as well as malignant human breast epithelial cells.

**Keywords:** normal breast; type I insulin-like growth factor receptor; reverse transcription – polymerase chain reaction; oestrogen; progesterone

The type I insulin-like growth factor receptor (IGFR-I) is highly homologous to the insulin receptor having a heterotetrameric structure consisting of two ligand-binding extracellular α-subunits and two transmembrane β-subunits containing a cytoplasmic tyrosine kinase domain (Ullrich and Schlessinger, 1990). The IGFR-I binds, in order of affinity, the insulin-like growth factors IGF-I, IGF-II and insulin to which the IGFs are structurally related (Cullen et al, 1991*a*). The IGFs are mitogenic for many cell types (Daughaday and Rotwein, 1989; Humbel, 1990).

In both normal and malignant human breast tissue, IGF-I and IGF-II mRNA are expressed by stromal fibroblasts, but generally not by the epithelium (Yee et al, 1989; Paik, 1992), whereas the expression of the IGFR-I is restricted to epithelial cells (Jammes et al, 1992). In vitro studies of the breast cancer cells have demonstrated that the IGFs are potent mitogens (Karey and Sirbasku, 1988), and that their growth-stimulatory effects can be blocked, both in vitro and in vivo, by a specific antibody (αIR-3) to the IGFR-I (Arteaga et al, 1989; Cullen et al, 1990). This suggests that growth, at least of MCF-7 breast cancer cells, is stimulated via the type I rather than the type II IGF receptor.

IGF-I, IGF-II or insulin in combination with $E_2$ are synergistic in their effects on the growth of MCF-7 cells in culture (Stewart et al, 1990; Thorsen et al, 1992). Significantly, the expression of the IGFR-I and its mRNA in MCF-7 breast cancer cells is greatly increased by $E_2$ treatment (Stewart et al, 1990), which may sensitize the cells to the mitogenic effects of the IGFs. The synergism between $E_2$ and IGF-I in MCF-7 cells can be abrogated by adding the αIR-3 antibody, which prevents binding of IGFs to the IGFR-I (Thorsen et al, 1992). In another breast cancer cell line, T47D, treatment with progesterone (Pg) was found to decrease the expression of the IGFR-I, whereas it had no effects on the growth of the cells (Papa et al, 1991).

Little is known about whether synergism between $E_2$ and the IGFs is important for the growth of the normal human breast. Studies of the steroid responsiveness of normal human mammary epithelial cells in culture have indicated mitogenic effects of $E_2$ (Malet et al, 1988) and growth-inhibitory effects of progesterone (Gompel et al, 1986). However, other studies have found no effect of $E_2$ upon human mammary epithelial cell proliferation in vitro (Richards et al, 1988; Gabelman and Emerman, 1992). In contrast, insulin, epidermal growth factor (EGF) and transforming growth factor-α (TGF-α) have been shown to be potent mitogens for normal breast epithelial cells in culture (Gabelman and Emerman, 1992; Perusinghe et al, 1992). We have found no reports of synergism between $E_2$ and the IGFs or insulin in normal human mammary epithelial cells in vitro. However, in vivo studies of rat mammary gland development indicate that IGF-I can substitute for growth hormone to elicit mammary development when administered together with $E_2$. Furthermore, $E_2$ treatment can sensitize the rat mammary gland to the local effects of IGF-I on development

*Received 18 June 1996*
*Revised 28 August 1996*
*Accepted 29 August 1996*

*Correspondence to:* RB Clarke

(Ruan et al, 1995). The suggestion that $E_2$ treatment may increase levels of the IGFR-I in vivo is further supported by data showing that $E_2$ treatment regulates IGFR-I expression in the rat uterus (Ghahary and Murphy, 1989).

The above evidence supports the suggestion that $E_2$ may be acting in the normal breast in a similar manner to that in human breast cancer cells in vitro where induction of the IGFR-I by $E_2$ may account for the synergistic effects of $E_2$ and IGF-I on growth. We have recently published a study which employed an in vivo model of normal human breast tissue implanted into athymic nude mice to study the effects of human adult serum concentrations of $E_2$ and Pg on epithelial proliferation and steroid receptor expression (Laidlaw et al, 1995). This study showed that $E_2$ at serum levels seen in the human menstrual cycle stimulated proliferation of the normal human mammary lobular epithelium in breast tissue implanted into nude mice in a dose-dependent manner. Furthermore, administration of Pg alone or in combination with $E_2$ had no effect. The objectives of the present investigation were to analyse the expression of the IGFR-I messenger RNA in the normal human breast xenografts treated with human serum concentrations of $E_2$ and/or Pg.

### MATERIALS AND METHODS

### Normal breast tissue implants

The normal breast tissue was obtained from patients undergoing surgery for isolated benign lesions, implanted subcutaneously into female athymic nude mice and treated with ovarian steroids using slow-release silastic pellets as described previously (Laidlaw et al, 1995). On excision from the nude mice, the normal breast xenograft tissue was frozen in liquid nitrogen until extraction of RNA. Six untreated human breast tumour samples were also obtained from patients at surgery in the course of studies on receptor expression and stored frozen in liquid nitrogen until used for assay.

### MCF-7 breast cancer cell culture

Approximately $10^6$ cells were plated in 2 ml of normal growth medium in 50-mm diameter wells. Normal growth medium was Dulbecco's modified Eagle medium (DMEM) (Gibco BRL, Paisley, UK) containing 10% fetal calf serum (Seralab, Crawley Down, Sussex, UK), 2 mM glutamine (Gibco BRL), 1 mM pyruvate (Gibco BRL), 50 units $ml^{-1}$ penicillin (Gibco BRL), 50 μg $ml^{-1}$ streptomycin (Gibco BRL) and 10 μg $ml^{-1}$ insulin (Hypurin, Baxter Healthcare, Manchester, UK). The cells were allowed to attach for 24 h and then withdrawn from the oestrogen and serum by culture for 4 days in withdrawal medium consisting of phenol red-free modified Eagle medium (Gibco BRL) containing 2 mM glutamine, 1 mM pyruvate, 50 units $ml^{-1}$ penicillin, 50 μg $ml^{-1}$ streptomycin, 25 μg $ml^{-1}$ transferrin and 10 μg $ml^{-1}$ Hypurin. Withdrawn cells were then cultured in either withdrawal medium alone or withdrawal medium supplemented with 0.1 nM, 1 nM or 10 nM $E_2$ for 5 days. The culture medium was changed on days 2 and 4 of treatment and the cells harvested on day 5. Each treatment was carried out in triplicate and RNA was extracted from these cells.

### RNA extraction

The cells were lysed by the application of 2 ml TRIzol reagent (Gibco BRL), a monophasic solution of phenol and guanidine isothiocyanate, and passaged through a pipette several times. Isolation of RNA from either frozen normal breast xenograft or breast tumour tissue was performed using at least 1 ml of TRIzol reagent per 50 mg of tissue. The frozen tissue was fragmented using a scalpel blade, placed in 1 ml of TRIzol reagent in an autoclaved microtube and homogenized. The RNA was then isolated according to the manufacturer's instructions and quantified by spectrophotometry at 260 nm. The RNA was stored as a precipitate in 0.3 M sodium acetate (pH 5.2), 70% v/v ethanol at –70°C. Owing to the small size of normal breast xenograft samples, several from each treatment group were pooled before the extraction of RNA for use in Northern blot analysis and quantitation by RT–PCR.

### Northern blot analysis

RNA (20μg) for Northern analysis was denatured and applied to 1.2% w/v agarose gels containing 2.2 M formaldehyde prepared and electrophoresed at 4 V $cm^{-1}$. RNA was transferred to positively charged nylon membrane by capillary action after partial hydrolysis of the RNA (Sambrook et al, 1989). Northern hybridization was performed by a standard technique (Stewart et al, 1994) using $\alpha$-$^{32}$P-labelled cDNA probes for either human IGFR-I (nucleotides 2438–2684; Ullrich, 1991) or human 36B4 (nucleotides 604–974; Rich and Steitz, 1987). The membranes were exposed overnight in a cassette against a storage phosphor screen followed by the scanning of the screen on the Molecular Dynamics 425S PhosphorImager. The image obtained was analysed using the Molecular Dynamics ImageQuant software.

### Oligonucleotide primers

Oligonucleotide primers were designed in order to amplify specific regions of cDNA sequences using the polymerase chain reaction (PCR), which could then either be cloned onto plasmid vectors or used for the quantification of sample mRNA expression. The cDNA sequence to be amplified was known to span at least one genomic intron by reference to the gene exon sequences to avoid amplification of any genomic DNA contaminants. The primers were manufactured on an Applied Biosystems (ABI, Warrington, Cheshire, UK) 394 DNA/RNA synthesizer using standard phosphoramidite chemistry and deprotected by incubation in ammonia at 60°C for 4 h. The solvent was evaporated by centrifugation under vacuum, after which the primers were dissolved in 1 ml of double autoclaved ultrapure water and quantified by their absorbance at 260 nm. The human 36B4 housekeeping control gene primers were designed by reference to the sequence for the human acidic ribosomal phosphoprotein PO (Rich and Steitz, 1987), since this has been shown to be identical in cDNA sequence (Laborda, 1991) to the 36B4 cDNA originally isolated by Masiakowski et al (1982). For the amplification of 36B4 cDNA, a forward primer (36B4-F1) consisting of nucleotides (nt) 604–621; 5′-ACATGCTCAACATCTCCC-3′ and a reverse primer (36B4-R1) consisting of nt 957–974; 5′-TTCAACCTTAGCTGGGGC-3′ were manufactured. For the amplification of human IGFR-I cDNA, a forward primer (IGFR-I-F1) consisting of nt 2438–2455; 5′-TGTACCGCATCGATATCC-3′ and a reverse primer (IGFR-I-R1) consisting of nt 2667–2684; 5′-ACACATTATCGCTGATCC-3′ were manufactured by reference to the published sequence (Ullrich, 1991).

© Cancer Research Campaign 1997

### Construction of synthetic standard cDNA

Synthetic standard cDNA was constructed by introducing a unique restriction enzyme site *Eco*RI into the wild-type cDNA to allow the separate quantitiation of wild-type and synthetic cDNA after PCR amplification, digestion and gel electrophoresis. This was facilitated by site-specific substitution of up to three nucleotide residues within the central portion of the DNA sequence to be amplified by PCR. For PCR-directed, site-specific introduction of an *Eco*RI restriction site within this amplified sequence, a further two 36B4 and IGFR-I primers were manufactured containing the *Eco*RI recognition sequence at their 5′ ends. These were: 36B4-F2, nt 791–806, 5′-CGGAATTCCCATTCTATCATCAAC-3′; 36B4-R2, nt 773–786, 5′-CGGAATTCTGATGCAACAGTT-3′; IGFR-I-R2, nt 2544–2557, 5′-CGGAATTCATTCCTGGGCCAG-3′; and IGFR-I-R2, nt 2527–2540, 5′-CGGAATTCTGCTCCTTCTGC-3′. The wild-type 370-bp (nt 604–974) 36B4 and 246-bp (nt 2438–2684) IGFR-I cDNA sequences were amplified by PCR using 36B4-F1 and 36B4-R1 primers, and IGFR-I-F1 and IGFR-I-R1 primers respectively. The amplified cDNA was cloned onto pCRII using the TA Cloning System (Invitrogen, San Diego, CA, USA), and the sequence of these wild-type cDNA clones as confirmed by dideoxy chain termination sequencing (Sanger et al, 1977). The *Eco*RI restriction sites were then introduced into these cDNA sequences by their PCR amplification using the following pairs of primers in separate tubes: 36B4-F1 and 36B4-R2; 36B4-F2 and 36B4-R1; IGFR-I-F1 and IGFR-I-R2; and IGFR-I-F2 and IGFR-I-R1. This yielded two cDNA products for each cDNA species, each cDNA fragment containing an *Eco*RI site at one end. These sites were then digested with the *Eco*RI restriction endonuclease (Promega, Madison, WI, USA), and the two PCR-generated fragments corresponding to either 36B4 or IGFR-I cDNA were mixed in a 1:1 ratio and ligated using T4 ligase (Promega). The 36B4 and IGFR-I cDNA sequences containing the unique *Eco*RI restriction site were amplified from the ligation reaction using the 5′ and 3′ flanking primers (36B4-F1 and 36B4-R1, IGFR-I-F1 and IGFR-I-R1 respectively). These synthetic standard 36B4 and IGFR-I cDNAs containing the *Eco*RI restriction site were then cloned onto pCRII using the TA Cloning System (Invitrogen). Sequencing by the dideoxy chain termination method (Sanger et al, 1977) confirmed that the cDNA was identical to the wild-type cDNA except for the *Eco*RI site generated by three nucleotide substitutions in the cDNA.

The plasmid clones containing the synthetic standard cDNAs were linearized with *Hind*III, gel purified, followed by phenol–chloroform extraction and quantified by spectrophotometric absorbance at 260 nm. The absolute number of cDNA templates was calculated using the molecular weight of the plasmid and the cloned 36B4 or IGFR-I cDNA, and Avogadro's number. A stock solution containing $1.0 \times 10^9$ molecules $\mu l^{-1}$ of each synthetic standard cDNA was made by dilution and stored at –70°C. This stock solution was used to make all further dilutions for quantitative PCR.

### Reverse transcription

The total RNA samples isolated from MCF-7 breast cancer cells and normal human breast xenografts were reverse transcribed into cDNA by incubation of 1 µg of heat-denatured RNA for 30 min at 42°C in a 20-µl reaction volume. The reaction contained 250 ng of random hexamer primers (Promega), 1 mM dNTPs (Promega), 2 µl of Moloney mouse leukaemia virus (MMLV) reverse transcriptase (Promega) and 1 × reverse transcriptase buffer (Promega). The reaction was terminated by incubation at 95°C for 5 min. The cDNA was stored at –70°C.

### Polymerase chain reaction (PCR)

The cDNA obtained by the reverse transcription was amplified by PCR using forward and reverse primers specific to the sequence. Each amplification reaction (100 µl) contained cDNA, 200 ng of each primer and 200 µM dNTPs (Promega) in *Taq* DNA polymerase buffer (Promega) containing 1.5 mM magnesium chloride. The reaction mix was overlaid with 50 µl of sterile liquid paraffin and denatured at 94°C for 5 min on a Hybaid (Teddington, Middlesex, UK) 'Omnigene' thermal cycler. The polymerization reaction was hot started to increase the specificity of the amplification by the addition of 2 units of *Taq* DNA polymerase (Promega) at the annealing temperature. The thermal cycler was programmed to perform cycles of primer extension for 1 min at 72°C, denaturation at 94°C for 30s and annealing at the lowest $T_m$ of the specific forward and reverse primers for 30s with the final cycle having a 10-min primer extension time. Where PCR products were to be labelled with α-$^{32}$P-dCTP, 2.5 µCi were added to each reaction. For each PCR amplification of cDNA, extraneous DNA contamination of the reagents was controlled by the inclusion in each experiment of a tube containing an equivalent dilution of a reverse transcription reaction, containing no RNA, in place of the cDNA sample, along with the same PCR reagents (minus cDNA control). The absence of amplified DNA in this –cDNA control confirmed that the reagents and their handling introduced no DNA contamination into the PCR tube. A second control tube for each cDNA sample contained the corresponding RNA sample that had not been reverse transcribed, along with the same PCR reagents (minus reverse transcriptase control; –RT). The absence of amplified DNA in this –RT control confirmed that the RNA extract was not contaminated with genomic or extraneous DNA.

### Quantitative PCR

A similar method for assaying mRNA species has been described previously (Backer-Andre and Hahlbrock, 1989) and employed synthetic standard RNA molecules at a range of concentrations alongside RNA extracted from tissue samples in the reverse transcription reaction followed by PCR amplification. In the present study, this has been modified by the use of synthetic standard cDNA molecules. The differences between samples in the quantity of RNA reverse transcribed into cDNA were internally controlled for by normalization against the expression of the house-keeping gene, 36B4. This modification of the previous method therefore controls for differences in the amounts of sample RNA and cDNA put into the assay, as long as the quantity of cDNA in the sample lies within the range of standard cDNA concentrations assessed. Approximately 1 µg of total cellular RNA from each sample to be analysed was reverse transcribed into cDNA. Equal quantities of sample cDNA, equivalent to 10 ng of reverse-transcribed total RNA, were amplified for 40 cycles of PCR, as described above, alongside a range of known quantities of the standard cDNA ($10^3$–$10^7$ molecules). For each quantity of the standard cDNA, the reaction mix contained either 36B4-F1 and 36B4-R1 primers or IGFR-I-F1 and IGFR-I-R1 primers and [α-$^{32}$P]-dCTP in order to label the DNA product. For each cDNA sample, a master mix of PCR reagents was prepared to ensure homogeneity of the

© Cancer Research Campaign 1997

constitutents between tubes, and appropriate minus cDNA and minus reverse transcriptase control tubes (as described above) were included. One caveat when using synthetic internal control cDNA containing the *Eco* RI restriction site is that the ratio of sample to standard may be affected by the formation of heterodimers between sample DNA and standard DNA strands. This was avoided by subjecting 1% of the PCR-amplified DNA product obtained after 40 cycles to one further cycle of PCR with fresh buffer and reagents. At this point, the reaction is once again in the exponential phase in which essentially every DNA molecule is amplified, thus forming only homodimeric DNA species (Backer-Andre and Hahlbrock, 1989). The reaction product was then digested with *Eco*RI, separated by polyacrylamide gel electrophoresis and phosphorimaged in order to quantify the relative amounts of amplified sample and standard cDNA. Polyacrylamide gels were prepared using 6% Long Ranger (AT Biochem, Malvern, PA, USA) gel solution. For each RNA sample, duplicate PCR amplifications were performed against a range of synthetic standard control cDNA concentrations from 1000 $\mu l^{-1}$ to $1 \times 10^7$ $\mu l^{-1}$. The ratio of sample–standard bands from each duplicate PCR amplification was calculated and the mean values plotted against the number of standard cDNA molecules in each reaction, through which a curve was drawn. The number of cDNA molecules in the sample is then equal to the point on the curve at which the ratio of sample–standard is one.

### RESULTS

### Northern blot analysis does not detect type I IGF receptor mRNA in total RNA extracted from normal human breast xenografts

Northern blot analysis confirmed that $E_2$ at doses of 0.1, 1 and 10 nM up-regulated the IGFR-I mRNA in MCF-7 breast cancer cells by two to threefold, and that it was expressed in breast tumour samples (data not shown). However, IGFR-I mRNA could not be detected in the normal breast xenografts by this method. This confirmed the findings of previous studies, which have indicated that the level of IGFR-I mRNA in normal breast tissue was either undetectable or lower than that in breast tumour tissue (Pekonen et al, 1988; Peyrat et al, 1990).

### Manufacture of standard cDNA by PCR-directed site-specific mutagenesis for use in quantitative RT–PCR

Since Northern blot analysis was not sufficiently sensitive to detect the IGFR-I mRNA in total RNA extracted from normal breast tissue xenografts, a quantitative RT–PCR strategy needed to be developed.

Standard cDNAs containing a single *Eco*RI restriction enzyme site not present in the natural cDNA sequence were manufactured for the regions of the human IGFR-I and 36B4 mRNAs, which would be amplified by the primers IGFR-I-F1 and IGFR-I-R1, and 36B4-F1 and 36B4-R1 respectively (see Materials and methods). A site approximately midway through each sequence was chosen, where an *Eco*RI recognition sequence could be created by substituting three nucleotide bases using a form of PCR site-directed mutagenesis, as described in the Materials and methods.

The PCR-amplified IGFR-I and 36B4 standard sequences were cloned onto a plasmid vector as described, which was cultured, purified, linearized by a single restriction enzyme cut within the plasmid polylinker, repurified and quantified by spectrophotometry at 260 nm. A stock solution containing $10^9$ molecules $\mu l^{-1}$ of both cDNA standards was then made. A range of known amounts of standard cDNA as prepared by diluting this stock for co-amplification alongside the endogenous cDNA that was reverse transcribed from cellular mRNA.

### Validation of the quantitative RT–PCR method for measuring IGFR-I gene expression using total RNA extracted from MCF-7 breast cancer cells

Approximately 1 µg of the total cellular RNA extracted from each sample to be analysed was reverse transcribed into cDNA. Equal quantities of the sample cDNA were then PCR amplified alongside a range of known quantities of the standard cDNA in a reaction mix containing [$^{32}$P] dCTP in order to label the DNA product. The reaction product was then digested with *Eco*RI, separated by PAGE and phosphorimaged in order to quantify the relative amounts of amplified sample and standard cDNA. The ratio of the sample–standard bands from each PCR amplification was calculated and plotted against the number of standard cDNA molecules added to each reaction. The number of cDNA molecules in the sample was presumed then to be equal to the point on the curve at which the sample–standard ratio was one. Since assessing the point at which the reaction was exponential for each sample would have been a lengthy process, this was avoided by subjecting 1% of the PCR-amplified DNA product obtained after 40 cycles to one further cycle of PCR with fresh buffer and reagents. At this point, the reaction was once again in the exponential phase in which essentially every DNA molecule was amplified ensuring the formation of homodimeric DNA species only.

A preliminary experiment was conducted in order to examine this phenomenon. MCF-7 IGFR-I and 36B4 cDNAs were separately amplified for 40 cycles in the presence of a range of standard cDNA concentrations. A sample of 1% of the DNA product was then subjected to a further cycle of PCR with fresh buffer and reagents and the product of this reaction was then digested with *Eco*RI. An equal proportion of the first PCR product with no extra cycle of amplification was also digested with *Eco*RI. This showed that the effect of one extra cycle of PCR did not greatly alter the estimation of the number of IGFR-I cDNA molecules, but reduced the estimation of the number of 36B4 cDNA molecules by more than half. This implied that the plateau phase of PCR for 36B4 cDNA amplification had been reached after 40 cycles, but that IGFR-I cDNA amplification had remained in the exponential phase. In all subsequent experiments, the dilution step followed by an extra cycle of PCR amplification was performed, so that the effects of the plateau phase on the formation of heterodimeric DNA species could be excluded.

In the final step of validation, the cDNA reverse transcribed from MCF-7 RNA was studied. MCF-7 sample cDNA, prepared from RNA extracted after either no treatment or treatment with 10 nM $E_2$, was separately amplified alongside a range of standard cDNA concentrations following the protocol for avoiding the formation of heterodimeric species. The phosphorimages obtained after digestion with *Eco*RI and separation by 6% polyacrylamide electrophoresis are shown in Figure 1A and B respectively. The ratios of sample–standard DNA product bands quantified by volume integration were calculated and plotted against the number of standard cDNA molecules (Figure 2A and B) and the point on the curve at which the ratio of IGFR-I or 36B4 equalled one was

© Cancer Research Campaign 1997

**Figure 1** The phosphorimages show the relative amounts of endogenous cellular cDNA amplified by PCR in the presence of increasing concentrations of standard cDNA. The samples analysed were cDNA reverse transcribed from (**A**) untreated MCF-7 breast cancer cell RNA and (**B**) 10 nM $E_2$-treated MCF-7 RNA. The amplified cellular and standard DNA bands were separated by PAGE on a 6% gel and the bands imaged after overnight exposure to storage phosphor screens. The resulting bands were quantified by volume integration of the phosphorimage

**Figure 2** The ratios of cellular to standard 36B4 or IGFR-I DNA obtained from the phosphorimage in Figure 1 were plotted as the mean of duplicates ± standard error (s.e.) against the initial standard 36B4 or IGFR-I cDNA concentration. At the point on the curve at which the ratio equalled 1:1, the sample IGFR-I or 36B4 concentration was read. The level of expression of IGFR-I mRNA in the initial sample was then calculated as a percentage of the expression of the housekeeping gene, 36B4. **A** shows the results obtained using RNA extracted and reverse transcribed from untreated MCF-7 cells, whereas **B** shows the results from cells treated with 10 nM $E_2$

**Figure 3** Summary of the data obtained by quantitative RT–PCR using a standard cDNA to estimate the IGFR-I mRNA expression in both MCF-7 and normal breast samples. For MCF-7 cells, each bar shows the amount of IGFR-I mRNA expressed as a percentage of that found for untreated cells. All RNA samples used were pooled from several experiments and analysed in duplicate. The column representing the results from xenografts treated for 7 days with $E_2$ has an error bar, which is the result of analysis of three separate pools of xenograft samples from which RNA was extracted read from the *x*-axis. As a further control for RNA loading and the efficiency of the RT reaction, the amount of IGFR-I cDNA initially present in the sample was calculated as a percentage of the amount of 36B4. The untreated sample contained IGFR-I cDNA at 5.6% of the levels of 36B4 cDNA, whereas the 10 nM $E_2$-treated sample contained IGFR-I cDNA at 15.6% of the levels of 36B4. This indicated a 280% increase in IGFR-I mRNA levels (Figure 3) and was in agreement with the increase detected by Northern analysis.

### Measurement of the effects of $E_2$ and Pg treatment on IGFR-I mRNA expression in normal human breast tissue xenografts by quantitative RT–PCR

The pooled xenograft sample cDNAs were analysed according to the RT–PCR methodology developed and validated as above. The xenograft samples were either untreated, treated with a 4 mg Pg pellet for 7 or 14 days, treated with a 2 mg $E_2$ pellet for 7 or 14 days, or treated with a combination of 2 mg of $E_2$ for 14 days combined with 4 mg of Pg for the final 7 days. The data obtained using this quantitative RT–PCR method indicated that 7 and 14 days' Pg treatment decreased IGFR-I mRNA levels to 30% and 60%, respectively, of the levels found in untreated xenografts (Figure 3). However, increases in IGFR-I gene expression of 250% (mean of analysis of three different pools of xenograft RNA

© Cancer Research Campaign 1997

samples) and 230% were observed after 7 and 14 days' treatment of xenograft with 2 mg of $E_2$ compared with no treatment (Figure 3). In those treated with the combination of $E_2$ and Pg, an increase in IGFR-I gene expression of 200% compared with no treatment was observed (Figure 3).

### DISCUSSION

In the study described above, we have shown that the normal breast expresses levels of the IGFR-I mRNA that are undetectable by Northern blot analysis, whereas the IGFR-I mRNA expression in breast cancer cells and tissue was easily detected. We have, therefore, developed a method of RT–PCR in order to quantitate changes in IGFR-I mRNA levels occurring when normal breast tissues implanted into nude mice were treated with human luteal phase serum concentrations of $E_2$ and/or Pg. We have previously published evidence that proliferation is controlled by $E_2$ in this model (Laidlaw et al, 1995), and we wished to determine whether changes in growth factor receptor expression could partly account for this mitogenic effect of $E_2$.

The method of quantitative RT–PCR that we have developed internally controlled for the different efficiencies of reverse transcription between extracted RNA samples, since the IGFR-I cDNA levels were normalized against levels of the constitutively expressed house-keeping gene, 36B4, cDNA. Expression of the 36B4 house-keeping gene has been widely used for normalization of RNA levels as assessed by RNAase protection assay both in breast cancer and in normal cells in vitro (Bronzert et al, 1987; Cullen et al, 1991*b*), and in breast cancer xenografts in vivo (Brunner et al, 1993), since it was first isolated as a gene the expression of which was unaffected by $E_2$ treatment of MCF-7 breast cancer cells (Masiakowski et al, 1982). The product of the 36B4 gene has subsequently been shown to be identical to a ribosomal protein whose mRNA is constitutively expressed (Laborda, 1991; Krowczynska et al, 1989). The ratio between IGFR-I and 36B4 mRNA in the cellular sample should be equivalent to that seen after reverse transcription into cDNA, assuming that the efficiency of the reverse transcription of the IGFR-I mRNA and the 36B4 mRNA into their respective cDNAs was equivalent. Since the primers used in the reverse transcription were random hexamers, there seems to be no reason why this should not have been the case. The present method differs from the original methods described for the quantification of cellular mRNAs (Backer-Andre and Hahlbrock, 1989; Wang et al, 1989) in that the present study used known quantities of a standard cDNA, whereas these investigators used known quantities of a standard cRNA. Our method is similar to that described by Gilliland et al (1990) and has the advantage that the prepared standard cDNAs are stable over many months of storage with little degradation, whereas RNA is easily degraded (Sambrook et al, 1989). In contrast to the method of Gilliland et al (1990), however, the quantification of an endogenous cellular control gene mRNA, such as the house-keeping gene, 36B4, has been carried out in order to control internally for the amount of cDNA reverse transcribed from the cellular mRNA. This has advantages over the previously described studies, which require equivalent amounts of each sample mRNA to be reverse transcribed in order to compare the levels of a particular mRNA between samples. The present assay method controls internally for differences in the amounts of sample RNA and cDNA, and suggests that it could be applied to RNA samples that are too small to be quantified by conventional means, for example, RNA extracted from single frozen sections, microdissected areas of sections or fine needle aspirates of cells from the normal breast in vivo.

The application of this method to the cDNA samples obtained by reverse transcription allowed quantitative assessment of the changes in IGFR-I mRNA expression in MCF-7 and normal breast xenograft samples treated with ovarian steroid hormones. In untreated MCF-7 cells, IGFR-I mRNA was calculated to be at 5.4% of the levels of 36B4 mRNA, whereas 5 days' treatment with 10 mM $E_2$ increased the level of IGFR-I to 15% of the levels of 36B4. This threefold increase is identical to the increase observed in IGFR-I levels by Northern blot analysis. A similar increase in IGFR-I mRNA expression was seen in the samples of normal tissue treated in vivo with $E_2$. In contrast, Pg treatment reduced IGFR-I mRNA levels to 30% and 60% of those in xenografts taken from untreated mice.

As far as we know, this is the first time that up-regulation of the IGFR-I mRNA by $E_2$ and its down-regulation by Pg in normal breast tissue has been reported. These findings are consistent with the observed up-regulation of IGFR-I mRNA observed in MCF-7 breast cancer cells treated with $E_2$ in our study and others (Stewart et al, 1990; Thorsen et al, 1992), and with the observed down-regulation of IGFR-I mRNA observed in T47D breast cancer cells treated with Pg (Papa et al, 1991). These previous studies of $E_2$ and Pg effects on IGFR-I showed that the changes in mRNA levels were reflected by equivalent changes in levels of the cell membrane receptor (Stewart et al, 1990; Papa et al, 1991). In the present study, we have not quantified the changes in cell surface IGFR-I concentrations. However, using [$^{125}$I]IGF-I hisoautoradiography (Jammes et al, 1992), we confirmed that the IGFR-I is expressed solely on the epithelial component of xenografted normal human breast tissue (data not shown). Although the IGFR-I mRNA levels measured by RT–PCR could be affected by changes in the ratio of epithelial to stromal cells in the xenografts, no change to this ratio in response to ovarian steroid administration was observed as assessed by histological examination of the xenografts (Laidlaw et al, 1995).

Since the IGFR-I is the mediator of the mitogenic response to IGFs (Cullen et al, 1990), its up-regulation by $E_2$ is likely to have biological significance in the normal breast. The IGFs are secreted by adjacent normal human breast stromal fibroblasts (Yee et al, 1989; Cullen et al, 1991*b*; Paik, 1992). This secretion may itself be increased by $E_2$ through the paracrine actions of $E_2$-stimulated epithelial secretion of platelet-derived growth factor (PDGF), which stimulates breast fibroblasts to secrete IGFs (Bronzert et al, 1987), or through the synergistic action of $E_2$ with growth hormone on breast fibroblast IGF secretion, as reported in the rat mammary gland (Ruan et al, 1995). IGFs are also available to the normal breast epithelium via an endocrine pathway, since IGF-I is secreted by the liver throughout adult life, although at higher serum concentrations in younger than older women as its secretion is related to growth hormone levels (Clemmons and Van Wyk, 1984). Thus, the sensitivity of the normal breast tissue to mitogenic stimulation by $E_2$ in a particular woman may be partly dependent on her circulating levels of IGF-I, and possibly IGF-II, as well as the up-regulation of the IGFR-I within the epithelium of the mammary gland.

In conclusion, the present study has indicated that the type I IGF receptor mRNA is expressed in normal breast tissue, but at a lower level than in breast cancer cells in vitro, or in breast tumour tissue. The mRNA expression of the IGFR-I gene could not be detected in normal breast xenografts by Northern blot analysis, although it was

© Cancer Research Campaign 1997

easily detected in breast cancer cells and breast tumour samples. A quantitative RT–PCR method has been developed to assess the effects of steroid hormone treatment on xenografts of normal breast tissue. This has produced data that indicate up-regulation of the IGF receptor mRNA by human luteal phase serum concentrations of $E_2$, and down-regulation of the receptor mRNA by human luteal phase serum concentrations of Pg, but only in the absence of $E_2$. In the context of knowledge about the actions of IGFs on breast cancer cells in vitro, an up-regulation of the type I IGF receptor by $E_2$ may lead to synergistic effects of $E_2$ and IGFs on normal breast epithelial cell growth in vivo. This combined action of $E_2$ and IGFs on cell growth suggests that exposure to both these mitogens could adversely affect the risk of developing breast cancer.

### ACKNOWLEDGEMENTS

We thank Mr Ian Laidlaw for his collaboration in developing the nude mouse model for steroidal treatment of normal human breast tissue and Dr Jenny Varley for critical reading of the manuscript.

### REFERENCES

Arteaga CL, Kitten LJ, Coronado EB, Jacobs S, Kull FC, Allred DC and Osborne CK (1989) Blockade of the type I somatomedin receptor inhibits growth of human breast cancer cells in athymic mice. *J Clin Invest* **84**: 1418–1423

Backer-Andre M and Hahlbrock K (1989) Absolute mRNA quantification using the polymerase chain reaction (PCR). A novel approach by a PCR aided transcript titration assay (PATTY). *Nucleic Acid Res* **17**: 9437–9446

Bronzert DA, Pantazis P, Antionades HN, Kasid A, Davidson N, Dickson RB and Lippman ME. (1987). Synthesis and secretion of PDGF by human breast cancer cell lines. *Proc Natl Acad Sci USA* **84**: 5762–5767

Brunner N, Yee D, Kern FG, Spang-Thomsen M, Lippman ME and Cullen KJ. (1993) Effect of endocrine therapy on growth of T61 human breast cancer xenografts is directly correlated to a specific down-regulation of insulin-like growth factor II (IGF-II). *Eur J Cancer* **29A**: 562–569

Clemmons DR and Van Wyk JJ. (1984). Factors controlling blood concentrations of somatomedin c. *Clin Endocrinol Metab* **13**: 113–143

Cullen KJ, Yee D, Sly WS, Perdue J, Hampton B, Lippman ME and Rosen N. (1990). Insulin-like growth factor receptor expression and function in human breast cancer. *Cancer Res* **50**: 48–53

Cullen KJ, Yee D and Rosen N. (1991*a*). Insulin-like growth factors in human malignancy. *Cancer Invest* **9**: 443–454

Cullen KJ, Smith HS, Hill S, Rosen N and Lippman ME. (1991*b*). Growth factor messenger RNA expression by human breast fibroblasts from benign and malignant lesions. *Cancer Res* **51**: 4978–4982

Daughaday WH and Rotwein P. (1989). Insulin-like growth factors I and II. Peptide, messenger ribonucleic acid and gene structures, serum and tissue concentrations. *Endocrine Rev* **10**: 68–91

Gabelman BM and Emerman JT. (1992). Effects of estrogen, epidermal growth factor, and transforming growth factor-α on the growth of human breast epithelial cells in primary culture. *Exp Cell Res* **201**: 113–118

Ghahary A and Murphy LJ. (1989). Uterine insulin-like growth factor-I receptors: regulation by estrogen and variation throughout the estrous cycle. *Endocrinology* **125**: 597–604

Gilliland G, Perrin S, Blanchard K and Bunn HK. (1990). Analysis of cytokine mRNA and DNA: detection and quantitation by competitive polymerase chain reaction. *Proc Natl Acad Sci USA* **87**: 2725–2729

Gompel A, Melet A, Spritzer P, Lalardrie JP, Kuttenn F and Mauvais-Jarvis P. (1986). Progestin effect on cell proliferation and 17β-hydroxysteroid dehydrogenase activity in normal breast cells in culture. *J clin Endocrinol Metab* **63**: 1174–1180

Humbel RE. (1990). Insulin-like growth factors I and II. *Eur J Biochem* **190**: 445–462

Jammes H, Peyrat J-P, Vilain M-O, Haour F, Djiane J and Bonneterre J. (1992). Insulin-like growth factor receptors in human breast tumour: localisation and quantification by histo-autoradiographic analysis. *Br J Cancer* **66**: 248–253

Karey KP and Sirbasku DA. (1988). Differential responsiveness of human breast cancer cell lines MCF-7 and T47D to growth factors and 17β-estradiol. *Cancer Res* **48**: 4083–4092

Krowczynska AM, Coutts M, Makrides S and Brawerman G. (1989). The mouse homologue of the human acidic phosphoprotein PO: a highly conserved polypeptide that is under translational control. *Nucleic Acids Res* **17**: 6408

Laborda J. (1991). 36B4 cDNA used as an estradiol-independent mRNA control is the cDNA for human acidic ribosomal phosphoprotein PO. *Nucleic Acids Res* **19**: 3998

Laidlaw IJ, Clarke RB, Howell A, Owen Awmc, Potten CS and Anderson E. (1995). The proliferation of normal human breast tissue implanted into athymic nude mice is stimulated by estrogen but not progesterone. *Endocrinology* **136**: 164–171

Malet C, Gompel A, Spritzer P, Bricout N, Yaneva H, Mowszowicz I, Kuttenn F and Marvais-Jarvis P. (1988). Tamoxifen and hydroxytamoxifen isomers versus estradiol effects on normal breast cells in culture. *Cancer Res* **48**: 7193–7199

Masiakowski P, Breathnach R, Bloch J, Gannon F, Krust A and Chambon P. (1982). Cloning of cDNA sequences of hormone-regulated genes from the MCF-7 human breast cell line. *Nucleic Acids Res* **10**: 7895–7903

Paik S. (1992). Expression of IGF-I and IGF-II mRNA in breast tissue. *Breast Cancer Res Treat* **22**: 31–38

Papa V, Hartmann KKP, Rosenthal SM, Maddux BA, Siiteri PK and Goldfine ID. (1991). Progestins induce down-regulation of insulin-like growth factor-I (IGF-I) receptors in human breast cancer cells: potential autocrine role of IGF-II. *Mol Endocrinol* **5**: 709–717

Pekonen F, Partanen S, Makinen T and Rutanen E-M. (1988). Receptors for epidermal growth factor and insulin-like growth factor I and their relation to steroid receptors in human breast cancer. *Cancer Res* **48**: 1343–1347

Perusinghe NP, Monaghan P, O'Hare MJ, Ashley S and Gusterson BA. (1992). Effects of growth factors on proliferation of basal and luminal cells in human breast epithelial explants in serum-free culture. *In Vitro Cell Dev Biol* **28A**: 90–96

Peyrat JP, Bonneterre J, Vennin PH, Beuscart R, Hecquet B, Djiane J, Lefebvre J and Demaille A. (1990) Insulin-like growth factor 1 receptors (IGF1-R) and IGF1 in human breast tumors. *J Steroid Biochem Mol Biol* **37**: 823–827

Rich Be and Steitz JA (1987). Human acidic ribosomal phosphoproteins PO, P1, and P2: analysis of cDNA clones, *in vitro* synthesis, and assembly. *Mol Cell Biol* **7**: 4065–4074

Richards J, Imagawa W, Balakrishnan A, Edery M and Nandi S. (1988). The lack of effect of phenol red or estradiol on the growth response of human, rat and mouse mammary cells in primary culture. *Endocrinology* **123**: 1335–1340

Ruan W, Catanese V, Wieczorek R, Feldman M and Kleinberg DL. (1995). Estradiol enhances the stimulatory effect of insulin-like growth factor-I (IGF-I) on mammary development and growth hormone-induced IGF-I messenger ribonucleic acid. *Endocrinology* **136**: 1296–1302

Sambrook J, Fritsch EF and Maniatis T. (1989). *Molecular Cloning: A Laboratory Manual*, Vol. 1–3. Cold Spring Harbor Laboratory Press: New York

Sanger F, Nicklen S and Coulson AR. (1977). DNA sequencing with chain-terminating inhibitors. *Proc Natl Acad Sci USA* **74**: 5463

Stewart AJ, Johnson MD, May Feb and Westley BR. (1990). Role of IGFs and the IGFR in the estrogen-stimulated proliferation of human breast cancer cells. *J Biol Chem* **265**: 21172–21178

Stewart JP, Behm FG, Arrand JR and Rooney CM (1994) Differential expression of viral and human interleukin-10 (IL-10) by primary B cell tumors and B cell lines. *Virology* **200**: 724–732

Thorsen T, Lahooti H, Rasmussen M and Aakvaag A. (1992). Oestradiol treatment increases the sensitivity of MCF-7 cells for the growth stimulatory effect of IGF-I. *J Steroid Biochem Mol Biol* **41**: 537–540

Ullrich A. (1991). Insulin-like growth factor I receptor cDNA cloning. *Methods Enzymol* **198**: 17–26

Ullrich A and Schlessinger J. (1990). Signal transduction by receptors with tyrosine kinase activity. *Cell* **61**: 203–212

Wang AM, Doyle ME and Mark DF (1989) Quantitation of messenger RNA by the polymerase chain reaction. *Proc Natl Acad Sci USA* **86**: 9717–9721

Yee D, Paik S, Lebovic GS, Marcus RR, Favoni RE, Cullen KJ, Lippman ME and Rosen N (1989) Analysis of insulin-like growth factor I gene expression in malignancy: evidence for a paracrine role in human breast cancer. *Mol Endocrinol* **3**: 509–517

© Cancer Research Campaign 1997